# A management model for admission and treatment of pediatric trauma cases

**DOI:** 10.1186/s13584-021-00506-5

**Published:** 2021-12-13

**Authors:** Raya Tashlizky Madar, Avishay Goldberg, Nitza Newman, Yehezkel Waisman, David Greenberg, Bruria Adini

**Affiliations:** 1 Nursing Academy, Barzilai University Medical Center, Ashkelon, Israel; 2 Department of Health Systems Management, Faculty of Health Sciences, Ben-Gurion University of the Negev, Beer Sheba, Israel; 3 PREPARED Center for Emergency Response Research, Ben-Gurion University of the Negev, Beer Sheba, Israel; 4 Pediatric Surgery Department, Soroka University Medical Center, Beer Sheva, Israel; 5 Department of Emergency Medicine, Schneider Children’s Medical Center of Israel, Petah Tikva, Israel; 6 School of Continuing Medical Education, Sackler Faculty of Medicine, Tel-Aviv University, Tel-Aviv, Israel; 7 Pediatric Infectious Disease Unit, Pediatrics Department, Soroka University Medical Center, Faculty of Health Sciences, Ben-Gurion University of the Negev, Beer-Sheva, Israel; 8 Department of Emergency Management and Disaster Medicine, School of Public Health, Sackler Faculty of Medicine, Tel-Aviv University, Tel-Aviv, Israel

**Keywords:** Emergency services, Trauma, Children, Severe injury, Health policy, Pediatric trauma cases

## Abstract

**Background:**

Pediatric trauma, particularly major trauma cases, are often treated in less than optimal facilities by providers who lack training and experience in treating severely injured children. We aimed to develop a management model for admission and treatment of pediatric trauma using the Theory of Constraints (TOC).

**Methods:**

We conducted interviews with 17 highly experienced policy makers, senior nursing managers and medical managers in pediatrics and trauma. The interviews were analyzed by qualitative methods. The TOC was utilized to identify undesirable effects (UDEs) and core challenges, and to design a focused current reality tree (CRT). Subsequently, a management model for optimal admission and treatment of pediatric trauma was constructed.

**Results:**

The CRT was illustrated according to 4 identified UDEs focusing on lack of: (1) clear definitions of case manager in pediatric trauma; (2) uniform criteria regarding the appropriate site for admitting pediatric trauma, (3) standard guidelines and protocols for treatment of trauma cases and for training of trauma medical teams; and (4) standard guidelines for evacuating pediatric trauma patients. The management model for treatment and admission of pediatric trauma is based on 3 major elements: human resources, hospital policy concerning the appropriate emergency department (ED) for pediatric trauma patients and clear definitions regarding children and trauma levels. Each of the elements contains components that should be clearly defined in order for a medical center to be designated for admitting and treating pediatric trauma patients.

**Conclusions:**

Our analysis suggests that the optimal ED for pediatric trauma cases is one with available operating rooms, intensive care beds, an imaging unit, laboratories and equipment suitable for treating children as well as with staff trained to treat children with trauma. To achieve optimal outcomes, medical centers in Israel should be classified according to their trauma treatment capabilities and their ability to treat varied severities of pediatric trauma cases.

**Supplementary Information:**

The online version contains supplementary material available at 10.1186/s13584-021-00506-5.

## Background

Trauma is the most common cause of pediatric mortality and disability globally [1, 2]. Differences in the mechanisms and patterns of injury observed in early childhood, late childhood, and adolescence, coupled with immature anatomic features and under-developed physiologic functions of pediatric patients, necessitate a unique response to major trauma and allocation of specialized pediatric resources [3]. Effective care of injured children requires a comprehensive and inclusive approach that recognizes childhood injury as a major public health challenge, identifies effective strategies for prevention, improves systems of emergency medical care for children, and provides the highest quality of pediatric trauma care [3, 4]. The organization of a trauma care system can substantially reduce trauma-related morbidity and mortality [5, 6].

Significant variability exists among health care systems concerning management of admission and treatment of pediatric trauma. These include designated pediatric medical centers versus medical centers that have been adapted for treating children. In some cases, pediatric casualties are admitted to general medical centers where they undergo initial stabilization and are then transferred to pediatric medical centers [7]. Significant variability has also been identified concerning designated pediatric trauma training and experience among physicians and nurses working in hospital emergency departments (EDs) [8].

Israel has 6 level-I trauma centers and 14 level-II trauma centers [9]. The trauma units are affiliated to surgical divisions of their respective hospitals [10]. While most medical centers also consist of a pediatric ED (PED) that is managed independently of the general ED, there are no pediatric trauma centers, and all major pediatric trauma cases are treated in general EDs, often by medical teams trained to treat adults [11].

The main characteristic required of any hospital that cares for pediatric trauma patients is the allocation of resources necessary to provide for this population’s specialized needs. The Task Force for Children’s Surgical Care together with the American College of Surgeons and the Children’s Hospital Association developed the Children’s Surgery Verification Quality Improvement Program to describe the necessary resources in a facility providing surgical care to children [12]. These include 24-h availability of a pediatric surgeon, pediatric surgical specialists, pediatric anesthesiology, pediatric intensive care, medical pediatric specialties, and the ability to provide comprehensive care to pediatric trauma cases of all ages [13]. Although the Israeli National council for Trauma and Emergency Medicine has discussed the challenges and current gaps of treating pediatric trauma cases and recommended varied measures, these gaps remain.

We aimed to develop a model for optimal management of admission and treatment of pediatric trauma cases in the ED using the Theory of Constraints (TOC).

## Methods

### Study design and participants

A qualitative study was conducted among 17 highly experienced policy makers, senior nursing managers and medical managers in pediatrics and trauma who work in the Israeli Ministry of Health and in medical centers across Israel (2 trauma experts, 2 heads of PEDs, 2 heads of general EDs, 2 pediatric hospital directors, 2 general hospital directors, a senior general and pediatric emergency medicine physician, a senior pediatric surgeon, 2 senior nurses and 3 policymakers). The same participants were previously interviewed regarding their attitudes and perspectives on the provision of medical care to pediatric trauma casualties in EDs in Israel [14]. All interviewees signed an informed consent form for participation in the study and publication of the opinions expressed in the interviews. The study was reviewed and approved by the institutional ethics committee.

### Data collection tool

Data were collected using a semi-structured interview guide that was based on the findings of a previous qualitative analysis that was conducted among the same stakeholders [14] as well as on a survey disseminated among 843 physicians and nurses working in general EDs and PEDs of 22 medical centers across Israel [15]. The guide included reference to the organization of the trauma system, infrastructure, equipment, training, human resources, perception of the pediatric patient and his/her family and other issues raised by interviewees. The interview topics are presented in Table 1.

**Table 1:** Interview guide

Subject	Questions
Unit organization	In your opinion, what are the resources required in the ED for admitting and treating injured children according to various degrees of injury with an emphasis on moderate and major trauma? (personnel, specific skills, infrastructure of dedicated equipment, etc.)
	The literature describes the importance of early assessments. In your opinion, what are the elements that should be emphasized? (organization of trauma teams, protocols)
	From your experience, what is the infrastructure required for admitting injured children according to the severity of injury?
	In your experience, what is the best way to standardize protocols and procedures in the various hospitals? For example, for triage?
	The literature and our research findings so far, describe dangerous situations in which injured children are moved around the hospital. How can this situation be addressed considering the shortage in hospital staff? What do you think are the criteria for enabling these transfers? Are there procedures for such transfers?
Infrastructure and equipment	In your opinion, which hospital infrastructure is necessary to provide optimal care for children with moderate and major trauma? For example, imaging, laboratories, pediatric sedation and anesthesia rooms
	The literature describes deficiencies in preparedness of equipment in EDs which may affect quality of medical care. How can existing international guidelines be applied to hospitals in Israel?
Human resource	How can conflicts between ED teams and pediatric surgery teams, which often arise from availability and miscommunication, be solved?
	What is your position on the cooperation among the various caregivers of injured children?
Training	How can cardiopulmonary resuscitation courses (PALS, ATLS) be standardized?
	Do you think there is room in the ED for a physician’s assistant with a paramedic training? Do registered nurses with pediatric ED specialization need specific training?
	How can awareness of teams to treating pain in children during life-saving activities be raised by training staff?
Child’s family	A document published in 2004 by the United States American Academy of Pediatrics reported the importance of family involvement in decision-making and childcare even during resuscitation (in seriously injured children). What do you think about that? Do you see it implemented in Israel?
Additional issues	Do you think there are any other issues that were not raised in the interview that you would like to bring to our attention?

### Data collection and analysis

The interviews were conducted between June and September 2017. All interviews were transcribed and analyzed as previously described [14]. Briefly, the analysis included identification of themes and common features in the transcribed interviews followed by categorical analysis and classification of the text into text segments that served for creating a list of tags, representing a set of concepts conveyed in each interview (Additional file 1: Table S1). A thematic framework was developed based on pre-defined elements and themes that emerged from the familiarization stage [16]. Additional categories were added based on textual contents that were identified during the second reading.

We utilized the interview results and our previous findings to develop a Current Reality Tree (CRT) using the TOC. The TOC is a means of identifying and addressing complex problems and non-linear processes such as provision of health services in a systematic and comprehensive manner because it considers that processes are part of a larger, intertwined system [17]. The TOC requires a much more involved investigation into the relationship between several root causes of all failures of processes in the system. The one root cause that leads to the most undesirable effects (UDEs) is labeled as the core problem (constraint). This constraint prevents an organization from reaching its goal. The TOC change strategy produces more sustainable results because it considers other processes that may affect the root causes of constraints in the system [18].

Following the identification of the UDEs and constraints of pediatric trauma units, the CRT was constructed. This graphic management tool delineates the current status of the systems, enables the identification of problematic and fundamental phenomena, and indicates what must be changed in the organization.

Based on the understanding of the required components and constraints that were identified, a management model for optimal admission and management of pediatric trauma cases in EDs was established.

## Results

Following the interviews and their analysis, 4 UDEs were identified, as described below.

### UDE 1: Lack of uniform criteria regarding the appropriate site for admitting pediatric trauma patients

All study participants mentioned lack of uniform criteria for selecting the appropriate site for admitting pediatric trauma patients.

…Why [a general ED]? Is it closer to the operating rooms? It is more distant than pediatric intensive care. This child will most probably need pediatric intensive care… Why did he actually go there [to the general ED], besides from it being a common practice? Which is… one of the elements that most explains processes that you sometimes do not understand 'because that's what they always did, so we keep going.' (Interviewee 4).

Most of the respondents perceived that children with minor trauma should be treated at a PED, and that children with major trauma should be treated at a general ED.

[If] there's not some major active life-threatening issue here… it seems very clear to me that one should be treated in a PED. (Interviewee 1)

Regarding major trauma a decision was made. The Council for Trauma and Emergency Medicine has decided that at present they[children] should be admitted to adult shock rooms. (Inteviewee 12)

Diverse perceptions were presented concerning the optimal sites for treatment of moderate trauma cases. A hospital director perceived that the policy regarding treatment of children with major trauma in a general ED stems from a long-lasting practice rather than an appropriate ‘informed’ decision. Two other participants expressed the view that the appropriate admission site for pediatric trauma should be defined locally by each hospital administration.

What's the benefit for children in a PED and in a general ED… Perhaps also to define or classify the extent of injury [for admission] to each ED. I assume that at both extremes the situation is clear. (Interviewee 4)

It really depends on the structure and working methods of each hospital. …one should be aware of all the advantages and disadvantages… and try to find the best option for managing major trauma in children. (Interviewee 17)

Some resources and infrastructure were noted as essential for determining the appropriate admission site, including the proximity of the ED to the operating room, the blood bank and the imaging unit (particularly to a computed tomography scanner [CT]).

Do we subordinate the admission of major pediatric trauma – I am not talking about minor or moderate trauma – to the PED? It’s clear that there is nursing and medical infrastructure, but it [the PED] is distant from the operating rooms, CT and the blood bank. (Interviewee 6)

The availability of human resources were also considered essential. The participants noted the importance of having surgeons from all subspecialties on call, particularly neurosurgeons and orthopedic surgeons.

There is accessibility to surgeons, orthopedists and anesthetists…these are the main professions that are central to major multi-system trauma. (Interviewee 4)

As for major trauma, it is clear to me that pediatric teams, pediatric surgeons should be involved. As for nurses,… I do not have a completely good answer. (Interviewee 1)

Hence, selection of an appropriate site for admitting pediatric trauma patients is constrained by the resources available within the ED, by the hospital’s infrastructure, and by the availability and expertise of human resources.

In the coming years I would leave [severe pediatric trauma] to the general ED so that a critical mass of people, and not just from the pediatric professions, would concentrate there. …today I cannot be sure that at any given moment I would have an available pediatric surgeon to treat the case. …with training and growing of teams. (Interviewee 6)

It's very clear to me. Children should be treated by someone who has the skills for treating children. …A child is a world of its own, with its own needs, with a family. It's not a small adult…. and that's the worldview of all services for children. (Interviewee 4).

Most participants perceived that due to the relative rarity of major trauma, specific medical centers should be designated as pediatric trauma centers to improve trauma teams’ expertise.

One participant noted that he does not believe that such a significant change in policy is feasible in Israel, while another commented that such a decision should be accompanied by a directive from the Ministry of Health.

There are only a few seriously injured children… the ideal would be to establish a pediatric center, but there is no chance. …it would never happen in Israel… …it would harm the [health] system because that money will be at the expense of something else. (Interviewee 5)

The suggestion to treat all major trauma cases at a dedicated trauma center was opposed by one of the respondents who stated that such a policy shift would mean less exposure of ED teams to trauma, limiting their experience and hence their competencies and proficiency. Another participant was concerned that the time required to transfer an injured child to the designated trauma center may negatively impact both treatment and outcome.

An unstable injury is transferred to the nearest hospital… Unstable means 'let's stabilize him in the ED here. Then, transfer him’. (Interviewee 9)

…if you designate centers, then the staff at other hospitals would not have experience in treating these cases. (Interviewee 2)

### UDE 2: Lack of clear definitions of who should be appointed as case manager in pediatric trauma

We found no clear definitions regarding the preferable case manager in pediatric trauma cases. Most participants believed that this role should be performed by a surgeon because he/she can make decisions on performing invasive procedures.

I think what needs to be right is that a pediatric surgeon meets the trauma team at the patient's bed. He leads the treatment …he is responsible for this child until someone else accepts responsibility for him. (Interviewee 17)

…it has to be a surgeon who specializes in trauma. (Interviewee11)

In contrast, other participants claimed that the case manager should be a specialist in pediatric emergency medicine because most pediatric trauma cases do not require a surgical intervention.

Who manages the care of our moderately injured child?… and of minor injury? I do. I call an orthopedist, but I discharge him in the end. So who is the manager, not the one who discharges? He writes to me as a consultant. These are terribly confusing words: consultant and manager… There is no such thing as a consultant… A consultant is in the morning when an internal specialist goes to the surgical ward to give a consultation…But in the evening when you are called upon, then you are 100 percent responsible… (Interviewee 9)

Additionally, the ED physician is responsible for the patient and calls surgeons at his/her discretion. One respondent noted that although he agrees that a specialist in pediatric emergency medicine should be the case manager, most of these specialists do not have the expertise for dealing with trauma cases. Two participants believed that the case manager should be an anesthesiologist or a pediatric intensive care specialist.

Because we as surgeons, even pediatric surgeons, don’t know what to do when this child is unstable. Unless it is a pediatric surgeon who heads the department of pediatric surgery. But certainly not a first-year or a second-year intern who is on duty at night or a pediatric anesthetist. (Interviewee 5)

Another participant stated that each hospital should have the autonomy to decide who would be appointed as the case manager according to the team members’ capabilities and expertise.

Each hospital should do what is good for the facility. In one hospital it will be managed by a pediatric surgeon, in another… by adult surgeons, in another… by pediatric surgeons. (Interviewee 13)

Compensation, such as remuneration was suggested as a means for motivating and enhancing the commitment of the case manager.

Participants asserted that communication among team members must be improved. They agreed that communication is an essential element of teamwork especially in stressful situations such as treatment of trauma cases. They suggested that team members familiarize themselves with the work habits of their colleagues, conduct discussions and that mutual respect will be the basis for building trust among team members. Three participants stated that involvement of management and clear role-setting are essential for proper communication among team members.

Today people value hierarchy less. The intern does not respect you because you are the ED manager. He respects you because you know something he doesn’t know and you can teach him. (Interviewer 9)

Once you have the trust of teams that know each other… and they are used to working together, then the result is the same as in any team that know each other on a personal level: whom to trust, how much to trust. It must exist in major trauma. (Interviewee 14)

I think there should be a monthly discussion about trauma cases. (Interviewee 17)

Someone whom the hospital appoints, so he has more authority to set procedures and protocols for how the child would be treated… Once it comes from management… people will comply and it can help prevent confrontations. (Interviewee 12)

### UDE 3: Lack of standard guidelines

#### Lack of standard guidelines for working procedures

Most participants also described non-standardized working procedures and protocols. Some participants noted that it is the responsibility of the Ministry of Health to provide guidelines and quality measures about treatment of pediatric trauma patients.

A hospital director must establish procedures and thus there will be standardization. (Interviewee 11)

There are still no organized protocols on the subject. Hospital management needs to establish them. (Interviewee 3)

Each hospital should define teams for every issue—from who makes tea in the morning through to who performs surgeries on pediatric trauma cases. (Interviewee 13)

Most participants noted that hospital management should issue procedures for the trauma division defining essential parameters in a clear and consistent manner. Such procedures should relate to the patients’ age (the maximum age at which the patient would still be considered a child), severity of injury, structure and work procedures of ED teams.

…it makes no sense to take a 13-year-old boy who is already 1.7 m tall and say he is a small child and put him in a children’s hospital. Physiologically… in terms of drug calculations… he isn’t a child. Any classification should be for very small children, I do not know which age… (Interviewee 8)

…even a moderately injured person should be defined and where they are going to be [treated]…. This requires collaboration among the teams of the adult ED, the PED and the trauma unit. Together they should check the definitions of who is and who isn’t. (Interviewee 9)

I definitely think the population of major pediatric trauma is well defined and that they belong in the general ED, and that if someone cuts his hand he belongs in the PED. (Interviewee 17)

#### Lack of standard guidelines for staff training

The participants claimed that training is insufficient. Some recommended that ED teams undergo training courses on care of pediatric patients with major and moderate trauma. One participant suggested to establish a body that would be responsible for training ED teams.

Standardization is very important… regular procedures… are not being done and should be done. There should be a central training body in the country that would identify all physicians and nurses who are experts in pediatric trauma…. and would intensively train them according to the same standards, and afterwards would deal with their ongoing training. (Interviewee 12)

What needs to be increased is the issue of which ED nurses have taken PALS **[**Pediatric Advanced Life Support] and have knowledge in caring for children in intensive care. This will be resolved with the introduction of a child trauma activation procedure that is currently being written by the Ministry of Health. (Interviewee 6)

In addition to pediatric advanced life support and regular triage simulations two participants recommended rotations of general ED and PED teams, as well as rotations of pediatric surgeons among hospitals across Israel.

Pediatric surgeons from all hospitals will do annual rotations through the same designated centers.. (Interviewee 2)

…it means that they have to do rotations or work with children on a daily basis. …when I was an intern… I did a rotation in pediatric surgery, it was mandatory. Now it is not mandatory. …we lost skills, and the smaller the child, the more we are afraid. (Interviewee 5)

Some respondents suggested that the training should include pediatric medicine and pediatric intensive care.

…in many hospitals the three professions involved in pediatric trauma are pediatric emergency medicine, pediatric intensive care and pediatric surgery, are not always included in [trauma] training. Emphasis should be placed on trauma training in pediatric residencies. …this is very much emphasized in adult medicine: in surgery, orthopedics and emergency medicine. (Interviewee 8).

This lack of consensus may stem from the professions of the various leaders that were interviewed.

### UDE 4: Lack of standard guidelines for evacuating children with trauma

Conflicting perceptions were expressed concerning the proficiency of Magen David Adom (MDA), the Israeli emergency medical services (EMS) to decide the evacuation destination of pediatric trauma patients. MDA was described by the participants as an extremely organized EMS body that works according to clear procedures. Simultaneously, the participants noted that the decision to evacuate trauma patients to a specific medical center is often based on the paramedic’s intuition alone, rather than on substantiated competencies and knowledge.

If [MDA] send minor trauma to the PED, …one in… [cases] someone who was not supposed to get there will arrive…now you will run with him back to the general ED or you will start managing it in the PED without the staff and the dedicated equipment… and it might end badly. On the other hand, because of the possibility that one in a hundred [cases] will end badly, would I bring all ninety-nine other cases to the general ED? (Interviewee 2)

Additionally, the participants claimed that MDA teams are not exposed to many cases of major pediatric trauma, creating difficulty in treating such cases until they are admitted to the ED.

They have no skill. They feel. Think how many paramedics do shifts. The chance that the same paramedic would deal with much trauma is zero. (Interviewee 12)

Many participants also raised the issue of the high percentage of injured children that are evacuated by their families to PEDs rather than to the general EDs.

…quite a few have children that were evacuated by their families… …they usually bring the children to the PED rather than to the general ED. …we need to decide and to publicize it so that the population would know how and where to bring children. (Interviewee 6)

### Focused current reality tree and trauma model

**Fig. 1:**
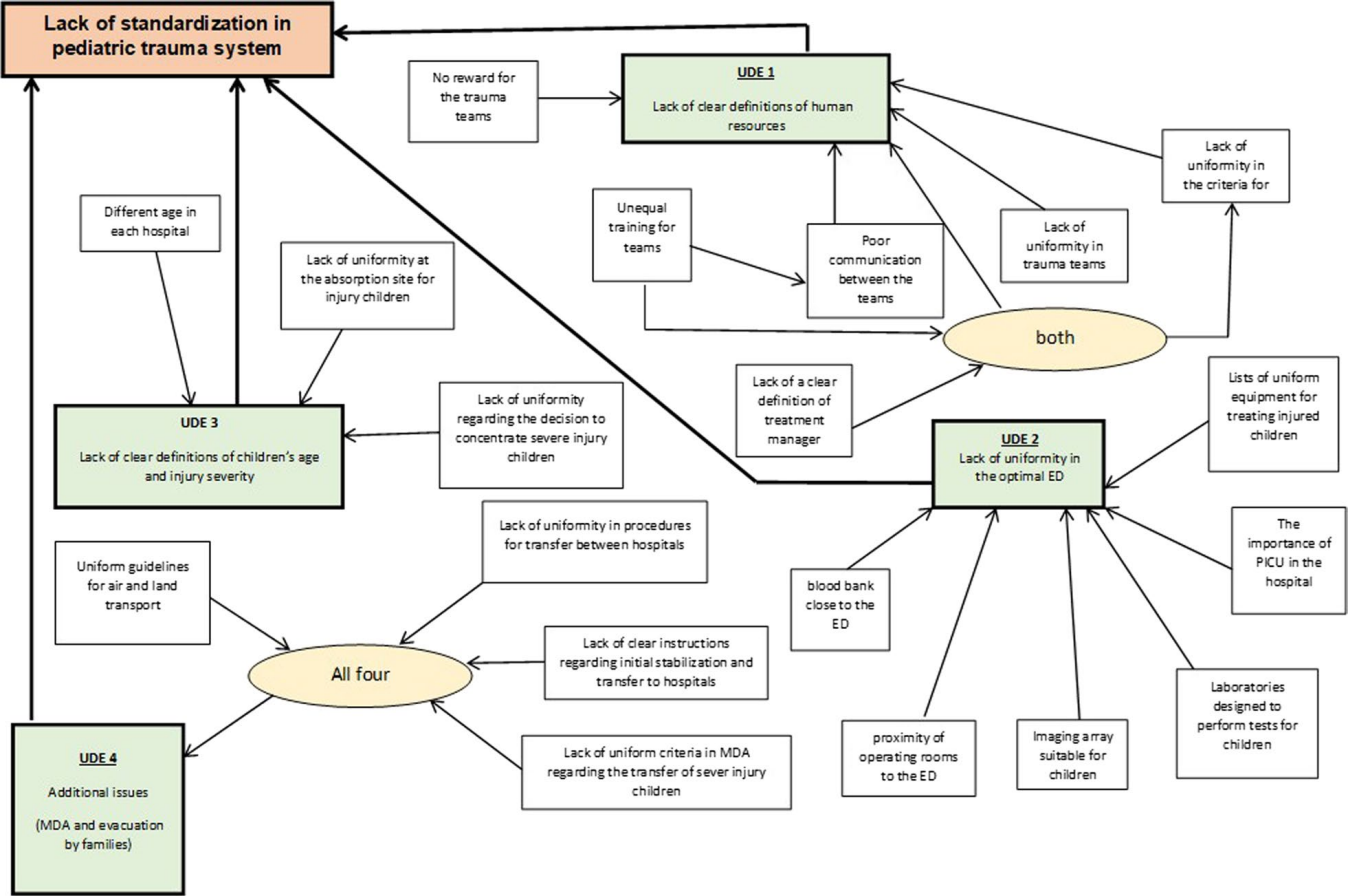
Focused current reality tree for management of admission and treatment of pediatric trauma cases. Abbreviations: ED, emergency department; MDA, Magen David Adom; UDE, undesirable effect

**Fig. 2:**
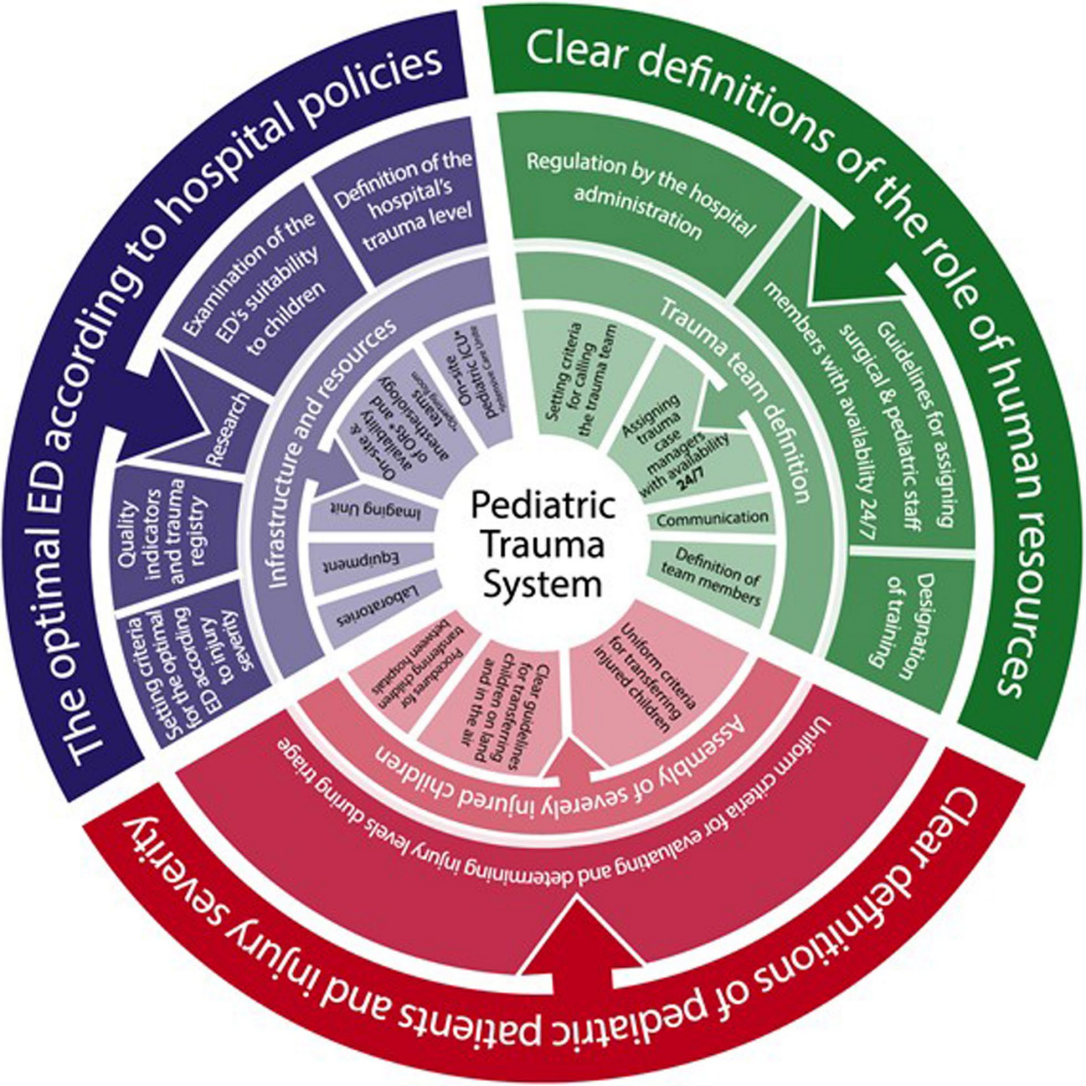
Model for optimal management of admission and treatment of pediatric trauma cases

After identifying the UDEs and constraints, a current reality tree was constructed (Fig. 1), and subsequently, a model for a trauma division for pediatric trauma was designed (Fig. 2). The model was founded on the "complete kit" concept, whereby children can be admitted to designated medical facilities, only after all the constraints in the system have been addressed and rectified. The model comprises 3 major elements: human resources, hospital policy concerning the appropriate ED for admitting and treating pediatric trauma patients, and clear definitions regarding pediatric trauma patients and required trauma levels. Each element contains components that should be clearly defined in order for a medical facility to be designated as a medical center, optimal management of admission and treatment of pediatric trauma patients. The model was endorsed by the National Council for Trauma and Emergency Medicine, that consists of leading experts in trauma management.

## Discussion

Our analysis revealed UDEs that are derived due to the lack of: clear definitions of which medical professionals should be appointed as case managers of pediatric trauma; criteria regarding the appropriate site for admitting pediatric trauma patients; standard guidelines; and standard guidelines for evacuating children with trauma. These issues are not unique to the Israeli healthcare system and have been described in other countries such as the United States [19].

Our analysis revealed that work procedures, definitions and protocols regarding the treatment of pediatric trauma patients should be improved. Lack of guidelines and protocols were also reported regarding other health systems [20, 21]. For example, there is no clear definition for the maximal age at which children would still be considered pediatric patients and treated based on pediatric protocols [14, 19, 22, 23]. An additional problem is the lack of standard definitions of injury levels, such as what constitutes minor, mild or severe injury, which would facilitate the decision on the appropriate site of admission and treatment.

The human resource, i.e., team composition and case manager, is one of the most significant and vital challenges of treating injured children [21, 24]. There is no scientific consensus regarding who should be the case manager of pediatric trauma cases. In Israel, children with major trauma are usually admitted to a general ED and are often treated by a general surgeon. However, to provide optimal care, especially during emergency situations such as trauma, children should be treated by practitioners who have specialized in their unique anatomy, physiology, and pathophysiology, using specialized equipment, and appropriate resources for potential hospitalization and surgical intervention [3, 25]. Studies have found mixed results regarding the impact of adult trauma surgeons treating pediatric trauma cases on mortality, time to the operating room and length of stay [26–29]. Our study also identified a lack of consensus among the interviewees regarding who should be appointed as the case manager of pediatric trauma cases. Most participants believed that this role should be performed by a surgeon because he/she can make decisions on invasive procedures. In contrast, several interviewees disagreed with the claim that pediatric trauma is a surgical disease as most injured children require conservative treatment without any surgical intervention [23, 30–32]. It has also been suggested that a pediatric emergency medicine specialist be assigned as case manager [8, 23]. Leeper et al. reported a difference in terms of the rate of misdiagnosed injuries between trauma team leaders with a surgical versus nonsurgical training background [32]. In a study that evaluated the variation in structure and care processes for critically injured children among institutions with pediatric intensive care units that treat trauma patients in the United States, Flynn-O’Brien et al. found that the primary decision maker for injured children in the trauma bay was most often the surgical attendant, irrespective of trauma team composition, hospital-type, and sub-specialty surgical training. An interesting view of one participant was that each hospital should have the autonomy to decide who would be appointed as the case manager according to the team members’ capabilities and expertise, which may be acceptable due to the shortage of experienced personnel.

The shortage of pediatric emergency medicine specialists constitutes a significant risk to the quality of care provided to severely injured children [8, 23, 24]. In Israel, there is a shortage of trauma surgeons. Most medical centers in Israel have only one general surgeon who specializes in trauma. These circumstances expose injured children who require immediate surgical intervention to risk [33].

An additional UDE that was identified is the lack of clear and uniform guidelines regarding the appropriate ED to which pediatric trauma cases should be admitted. Studies have shown better treatment outcomes in designated trauma centers for children and in integrated trauma centers, compared to adult trauma centers [34–38]. Compared to children who were treated in general hospitals, children who were treated at designated pediatric centers, were treated conservatively, needed less surgical interventions, required less hospitalization days and the economic expenditure was lower [34, 39]. Designated pediatric trauma centers provide their trauma staff with more exposure to major trauma, enhancing their confidence in treating such cases, and increasing their professional capabilities [40, 41]. Lower mortality rates were found in level 1 trauma centers with a large patient volume [9, 42].

Conversely, treatment of major trauma in designated centers may mean that time is lost when transferring the child to such a center and may impact treatment outcome. According to a report by “Beterem – Safe Kids Israel”, the distribution of deaths, ED admissions and hospitalization of children following unintentional injury varies by geographical region. Between 2008 and 2015, mean child mortality rate due to unintentional injury was 4.6 deaths per 100,000 children. These rates were the highest in the Southern and Northern districts of Israel (6 and 5.3 deaths per 100,000 children, respectively) and lowest in the Tel Aviv and Central districts (2.7 and 3.5 deaths per 100,000 children, respectively). Between 2012 and 2014, mean ED admission rate was 770 per 100,000 children; the highest rate of ED asmissions was in the Northern district (1,016.6 per 100,000 children) and lowest—in Judea and Samaria (367.4 per 100,000 children). During the same period, the mean rate of hospitalization due to unintentional injury among children was 82.9 per 100,000 children; the highest rates of hospitalizations due to unintentional injuries were highest in the Northern and Haifa districts (125.3 and 106.4 admissions per 100,000 children, respectively) and lowest in the Jerusalem and the Judea and Samaria districts (43.3 and 40.9 admissions per 100,000 children, respectively) [43]. Therefore, although most participants in the current study perceived that due to the relative rarity of major trauma and in order to improve trauma teams’ expertise, only specific medical centers should be designated as pediatric trauma centers to improve trauma teams’ expertise, this may not be feasible in Israel.

All participants agreed that the most appropriate ED for treating pediatric trauma is the one that can provide the best possible response to the needs of the children, equipped with the necessary infrastructure, including the proximity of the operating rooms, laboratories, blood bank, imaging system and CT unit. This is in accordance with previous reports that also emphasized the need for dedicated equipment and drug treatment [24, 31, 44].

The lack of working procedures and protocols regarding the issue of designated pediatric trauma centers, directly affects both primary care providers in the field (MDA) who are responsible for evacuating the injured children to the appropriate site for optimal treatment, and families who usually bring their children to the closest ED. Our interviewees referred to MDA because it is the largest emergency medical service in Israel; however, other smaller ambulance/emergency medical services are available in different regions of the country and these may have other working procedures or protocols regarding evacuation of injured children.

## Limitations

This study was based on the views and opinions of 17 participants. Although all interviewees are leaders in their respective fields and were considered as most suitable for reviewing the positions and understanding the systemic constraints, it is possible that additional interviews would allow for additional insights. To minimize this effect, validating techniques from the qualitative research world were used.

## Conclusions and policy implications

The analysis performed in the current study and a previous study enabled us to obtain an extensive image of the challenges of admission and treatment of injured children in EDs in Israel and to construct a model that would provide solutions to these challenges. The model was based on focused management theories and provides applicable solutions to improve pediatric trauma care in Israel.

The recommendations for establishing optimal conditions for treating pediatric trauma are listed in Table 2. It is extremely important to define children’s ages and trauma severity as a basis for managing their treatment. As major trauma in children is a relatively rare occurrence, establishing level I pediatric trauma centers in Israel may not be feasible. Therefore, we suggest considering which of the medical centers across Israel could serve as pediatric trauma centers by classifying them according to their trauma treatment capabilities, i.e., by evaluating the available human resources, infrastructure and equipment. This would allow improved quality of care as well as increase the exposure and expertise of trauma teams caring for severely injured children.

**Table 2:** Recommendations for establishing optimal conditions for treating pediatric trauma

The optimal ED	On-site pediatric intensive care unit
	On-site operating rooms and operating room teams
	On-site anesthesiology
	On-site equipment
	On-site imaging unit
	On-site laboratory
Clear definitions of the role of human resources	Setting criteria for calling the trauma teams
	Assigning trauma case managers with availability 24/7
	Communication program
	Definition of team members
Clear definitions of pediatric patients and injury severity	Uniform criteria for transferring injured children
	Clear guidelines for transferring trauma cases on land or by air
	Procedures for transferring children between hospitals

Our analysis suggests that the optimal ED for pediatric trauma cases is one with available and accessible operating rooms, intensive care beds, an imaging unit, laboratories and equipment suitable for treating children as well as with staff trained to treat children with trauma. It is of utmost importance to create a trauma registry and to perform research in level 1 and 2 trauma centers to identify vital lessons that can be learned.

The human resource was considered essential for pediatric trauma care. There is the lack of personnel who have expertise in treating pediatric trauma. Most medical centers in Israel have only one general surgeon who specializes in trauma. The interviewees in this study and previous ones have suggested that pediatric trauma case managers can be general or pediatric emergency medicine specialists, or pediatric or general surgeons [27, 28]. Regarding standardization of human resources thar treat pediatric trauma cases, each medical center should define trauma teams and available personnel for treating injured children 24/7. In addition, required training for trauma teams and means to implement the guidelines should be specified.

All medical and nursing teams working in provision of pediatric trauma care should undergo the same standard training, which should include rotations in medical centers experienced at providing pediatric trauma care. Attention should also be given to the lack of experienced staff in the public healthcare system, specifically during evenings, nights and on weekends. As treatment of injured children requires unique expertise and critical medical decision making at all times of day, medical teams working in this field should be remunerated.

Finally, although the Israeli emergency medical system is similar to other EMS entities in other developed countries, the Israeli system has unique characteristics that are derived from the structure of the Israeli healthcare system as well as the specific needs and risks of the population. Therefore, pediatric trauma care should be modified to the needs and characteristics of the Israeli population. To allow the best possible evacuation and subsequent treatment of injured children, evacuation procedures should be standardized among all emergency services across Israel and should be implemented across all levels of these organization—from the organizations’ managers to the drivers and paramedics working in the field.

The model constructed in this study was based on the perceptions and understanding of leaders in the field and experienced ED teams as well as on an in-depth analysis of the study’s findings. The model was endorsed by the Israel National Council for Trauma and Emergency Medicine.

The implementation of this study's findings and their integration in policymaking of pediatric trauma care in EDs may significantly improve the pediatric emergency medical services for the benefit of the children on the one hand, and the healthcare system on the other. Further studies should evaluate the implementation of this model and its effect on the outcomes of pediatric trauma patients.

## Supplementary Information

**Additional file 1: Table S1**. List of tags representing the set of concepts conveyed in the interviews

## Data Availability

The authors are not able to share the data in order to preserve the privacy of the respondents.

## Abbreviations

CRT: Current reality tree
CT: Computed tomography
ED: Emergency department
EMS: Emergency medical services
MDA: Magen David Adom
TOC: Theory of Constraints
UDE: Undesirable effects
