# Supplementary material for: A management model for admission and treatment of pediatric trauma cases
Source: Isr J Health Policy Res. 2021 Dec 13;10:73. doi: 10.1186/s13584-021-00506-5 (PMC8670149; doi:10.1186/s13584-021-00506-5)
Supplement: Supplementary file 1 — Additional file 1: Table S1. List of tags representing the set of concepts conveyed in the interviews [file 13584_2021_506_MOESM1_ESM.docx]

**Supplementary Table 1. List of tags representing the set of concepts conveyed in the interviews**

| **Tags** |
| --- |
| Lack of uniformity in trauma teams |
| Poor communication among teams |
| Lack of uniformity in the criteria for trauma teams |
| No reward for trauma teams |
| Unequal training for teams |
| Lack of a clear definition of treatment manager |
| Lists of uniform equipment for treating injured children |
| The importance of a pediatric intensive care unit in the hospital |
| Laboratories designed to perform tests for children |
| Imaging unit suitable for children |
| Proximity of operating rooms to the emergency department |
| Proximity of blood bank to the emergency department |
| The age range of “child” is defined differently in each hospital |
| Lack of uniformity regarding admission of injured children |
| Lack of uniformity regarding the decision to treat severely injured children at a designated site |
| Uniform guidelines for air and land transport |
| Lack of uniformity in procedures for transfer between hospitals |
| Lack of clear instructions regarding initial stabilization and transfer to hospitals |
| Lack of uniform criteria of emergency medical services (Magen David Adom) regarding the transfer of severely injured children |
